# Silencing a Chitinase Gene, *PstChia1*, Reduces Virulence of *Puccinia striiformis* f. sp. *tritici*

**DOI:** 10.3390/ijms24098215

**Published:** 2023-05-04

**Authors:** Jia Guo, Ying Mou, Yuanxing Li, Qing Yang, Xue Wang, Haocheng Lin, Zhensheng Kang, Jun Guo

**Affiliations:** State Key Laboratory of Crop Stress Biology for Arid Areas, College of Plant Protection, Northwest A&F University, Xianyang 712100, China

**Keywords:** wheat, *Puccinia striiformis* f. sp. *tritici*, chitinase, BSMV-mediated host-induced gene silencing (HIGS)

## Abstract

Chitin is the main component of fungal cell walls, which can be recognized by pattern recognition receptors (PRRs) as pathogen-associated molecular patterns (PAMP). Chitinase in filamentous fungi has been reported to degrade immunogenic chitin oligomers, thereby preventing chitin-induced immune activation. In this study, we identified the chitinase families in 10 fungal genomes. A total of 131 chitinase genes were identified. Among the chitinase families, 16 chitinase genes from *Puccinia striiformis* f. sp. *tritici* (*Pst*) were identified, and the expression of *PstChia1* was the highest during *Pst* infection. Further studies indicated that *PstChia1* is highly induced during the early stages of the interaction of wheat and *Pst* and has chitinase enzyme activity. The silencing of *PstChia1* revealed that *PstChia1* limited the growth and reduced the virulence of *Pst*. The expression level of TaPR1 and TaPR2 was induced in *PstChia1* knockdown plants, suggesting that *PstChia1* is involved in regulating wheat resistance to *Pst*. Our data suggest that *PstChia1* contributes to pathogenicity by interfering with plant immunity and regulating the growth of *Pst*.

## 1. Introduction

Plants have evolved an innate immune system to protect themselves from microbial pathogens [1,2]. The system consists of two main parts. One is that plants use pattern recognition receptors (PRRs) on their cell surfaces to sense conserved signatures of invading pathogens, including microbe- or host-derived damage-associated molecular patterns (MAMPs/DAMPs), and trigger the first layer of immune responses, known as pattern-triggered immunity (PTI) [3,4,5]. The other is that plants recognize, directly or indirectly, multiple virulence factors of pathogens through the intracellular conserved nucleotide-binding domain (NB) of leucine-rich repeat (LRR)-containing receptors (NLRs) and initiate effector-triggered immunity (ETI) [6]. The conserved or characteristic structure of microorganisms, which are known as MAMPs or pathogen-associated molecular patterns (PAMPs), can be recognized by plant PRRs, triggering PTI and causing a series of downstream immune responses, such as Ca^2+^ influx, reactive oxygen bursts, activation of mitogen-activated protein kinase (MAPK) cascades, massive transcriptional re-editing of immune-related genes, etc. [7,8,9]. PAMPs/MAMPs have been identified in bacteria, fungi, and oomycetes, such as flagellin, EF-Tu, and cold shock proteins from bacteria, transglutaminases (TGases), cellulose-binding elicitor lectin (CBEL), and elicitins from oomycetes, and xylanase EIX from fungi [10,11,12]. The typical PAMPs are flg22, a highly conserved 22-aa fragment of flagellin, and elf18, an 18-aa domain of EF-Tu, among others [10]. The flg22 activates the immune responses in *Arabidopsis* and other plants, and elf18 is thought to be a PAMP in *Brassicaceae* species [13]. Additional recent research suggests that chitin is a PAMP that induces PTI in plants [14,15,16].

Chitin is widely found in insect exoskeletons, crustacean exoskeletons, and fungal cell walls and is a polysaccharide consisting of N-acetyl-D-glucosamine [17]. As a molecular model for identifying potential pathogens, chitin participates in the innate immune system of animals and plants [18,19,20]. A number of studies have indicated that chitin fragments can act as PAMPs to induce innate immunity in many plants. Chitinase catalyzes the degradation of β-1,4 glucoside bonds in chitin. Chitinase plays an important role in the growth and development of arthropods, insects, and fungi, for example, in the remodeling of the cell wall structure in fungi [21]. Although chitin is not present in plants, chitinase in plants has different functions than in animals and fungi [22]. In the interaction between plants and pathogens, plants have a variety of defense mechanisms to guard against pathogen invasion, including pathogenesis-related (PR) proteins [23]. Chitinase is a subgroup of PR proteins. Plant chitinases are divided into PR-3, PR-4, PR-8, and PR-11, which hydrolyze exposed fungal cell walls, destroy the integrity of fungal cells, and release chitin oligomers to trigger plant immunity [24]. Another classification criterion for chitinases is the classification as glycoside hydrolases 18 and 19 (GH18 and GH19) based on the characteristics of their catalytic domains. GH19 is mainly found in plants. Unlike GH19, GH18 is widely distributed in bacteria, fungi, yeast, viruses, plants, and animals [25]. In plant and fungal coevolution, fungi have also evolved strategies to avoid chitin-triggered immunity in a defense response [26]. For instance, the cocoa pathogen, *Moniliophthora perniciosa* has evolved a chitinase that is enzymatically inactive but retains substrate binding specificity and is able to prevent chitin-induced immunity by binding to immunogenic chitin fragments [27]. A class of effectors with chitinase activity (EWCAs) have been found in powdery mildew fungi (*Ascomycota erysiphales*), which can decompose chitin oligomers and alter the host plant’s recognition of chitin oligomers, thus avoiding chitin-induced immunity [28]. All in all, the hydrolyzed products of chitinase can be used as PAMPs, and the chitinase of pathogenic fungi can also be recognized as a PAMP to induce plant immunity [29,30]. Because chitinase plays an important role in the battle between plants and pathogens, the research on it is particularly significant.

Stripe rust, caused by *Puccinia striiformis* f.sp. *tritici* (*Pst*) is one of the most harmful wheat diseases in the world [31]. As obligate biotrophs, *Pst* cannot be isolated and cultured and lacks a reliable transformation system. Its life history is very complex, and it has characteristics of transparasitism. Wheat stripe rust mutates in the natural environment and constantly produces new isolates, which can result in the loss of disease resistance of wheat varieties. Therefore, it is important to explore the interaction mechanism between wheat and the stripe rust fungus to enable the breeding of new resistant varieties. In this study, starting with the chitinase family of wheat stripe rust, we analyzed the difference in chitinase expression and enzyme activity before and after the infection of stripe rust. After silencing the chitinase family genes by the BSMV-mediated host-induced gene silencing (HIGS) technique, we measured the changes in the expression of genes related to wheat resistance. Our results contribute to our understanding of the action of chitinase in *Pst* and lay a foundation for exploring the molecular mechanism of interaction between wheat and stripe rust. 

## 2. Results

### 2.1. Identification of Chitinase Genes in 10 Fungal Genomes

To identify the chitinase family, 10 fungal genomes were selected, five from Ascomycota and five from Basidiomycota (Table S1). Hidden Markov models (HMMs) of seeds of GH18 (PF00704) and GH19 (PF00182) were submitted to search against those fungal genomes by HMMER3.0. In total, 142 putative genes were found. All the sequences were submitted to the HMMER database to confirm that the GH18 domain is contained. Finally, a local Blast was performed to remove the redundancy sequence, and 131 chitinase genes were identified, including 2 from *Saccharomyces cerevisiae*, 13 from *Sclerotinia sclerotiorum*, 18 from *Fusarium graminearum*, 16 from *Magnaporthe oryzae*, 19 from *Verticillium dahliae*, 4 from *Ustilago maydis*, 10 from *Uromyces fabae*, 16 from *Puccinia striiformis* f.sp. *tritici*, 17 from *Puccinia triticina*, and 16 from *Puccinia graminis* f.sp. *tritici* (Tables S1 and S2). 

**Figure 1:**
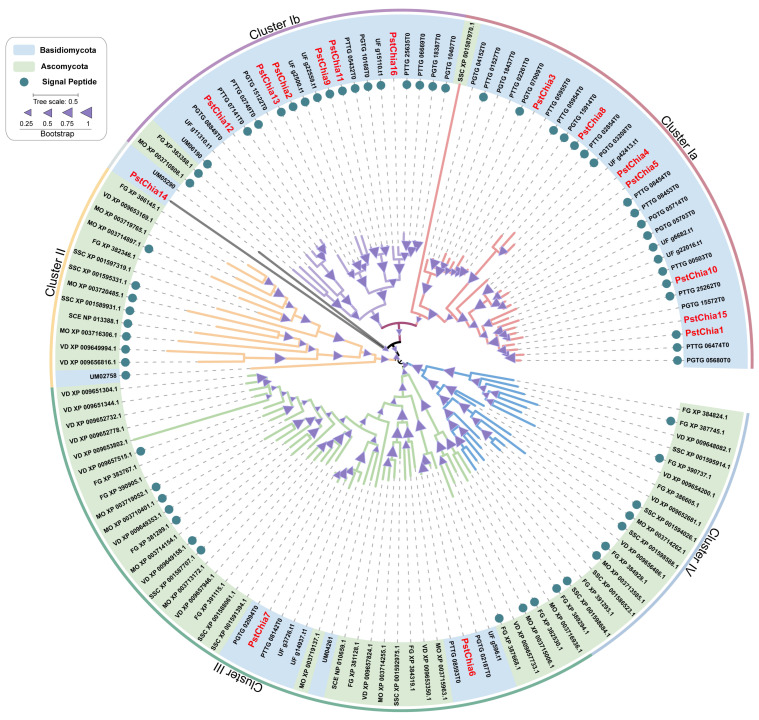
Phylogenetic relationship of the chitinase families. MEGA6 software was used to construct the maximum likelihood (ML) tree under the LG+F model. The Interactive Tree of Life (IToL) version 6.7.1 was used to draw the tree. The scale corresponds to 0.5 amino acid substitutions. The different clusters were labeled with different colors, and the chitinase family proteins with signal peptides were labeled with blue circles.

To investigate the phylogenetic relationships of the chitinase genes, a maximum likelihood (ML) phylogenetic tree was constructed from the 131 full-length amino acid sequences by MEGA software (Figure 1). All the chitinase genes were divided into four clusters (cluster I to IV). Cluster I was further divided into two subclusters, cluster Ia and cluster Ib, almost all of which were composed of chitinases in the Basidiomycota, except for SSC_XP_001587707.1 in cluster Ia and FG_XP_383388.1 and MO_XP_003710808.1 in cluster Ib. Cluster II and Cluster IV were primarily composed of chitinases in the Ascomycota, except for Um02758 in Cluster II. Cluster III contained a more conserved relationship with 10 chitinases from Basidiomycota and 34 from Ascomycota.

### 2.2. Expression Analysis of PstChias during Wheat-Pst Interaction

**Figure 2:**
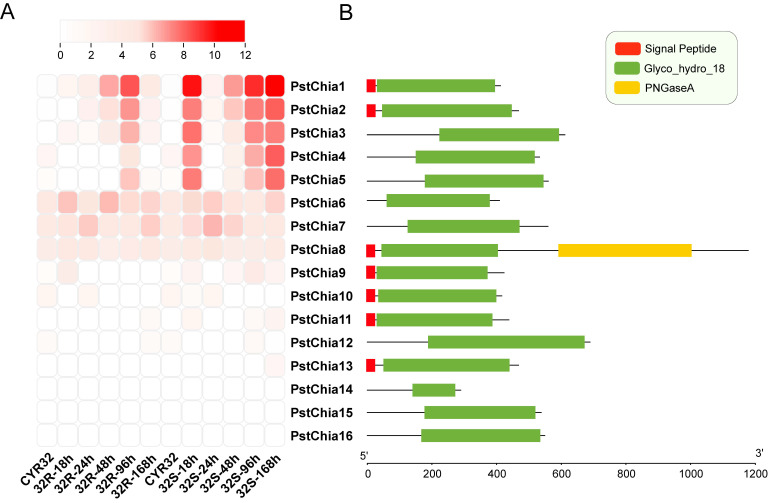
(**A**) The transcription levels of *PstChias* in the affinity interaction between wheat and *Pst*. The expression patterns of *PstChias* were detected by logFC [log_2_ (foldchange)] at 0, 18, 24, 48, 96, and 168 hpi sites in compatible groups and incompatible groups using time series double RNA-seq data. 32R indicates an incompatible combination, and 32S indicates a compatible combination. The shade of red color indicates the level of upregulation multiple; the darker the red, the higher the upregulation expression multiple. White indicates a similar expression pattern as observed in simulated therapy. (**B**) A model of the domain composition in the amino acid sequence of *PstChias* is shown by TBtools.

For gene expression analysis, the transcription levels during the interaction of wheat and *Pst* of all the *PstChias* were analyzed by the time series dual RNA-seq data. Chitinases in *Pst* were renamed from *PstChia1* to *PstChia16* by using total TPM to sort from high to low (Table S3). As shown in Figure 2A, *PstChia1-5* was upregulated at most time points in group 32S of the compatible interaction, whereas in the incompatible interaction group 32R, *PstChia1-5* was upregulated most highly at 96 hpi. On the other hand, *PstChia6/7/8* were constitutively expressed at all time points in both compatible and incompatible combinations. In addition, the expression level of *PstChia9* to *PstChia16* was low during wheat-*Pst* interactions. 

The domain structure analysis revealed the different gene lengths and domains of the *PstChias* (Figure 2B). Even in the genes with similar expression levels, the structure of the *PstChias* differed. For example, *PstChia1*, *PstChia2*, and *PstChia3* have similar expression levels, although *PstChia3* does not contain the signal peptide. *PstChia6*, *7*, and *8* were constitutively expressed, although *PstChia8* contains a PNGaseA domain and signal peptide.

**Figure 3:**
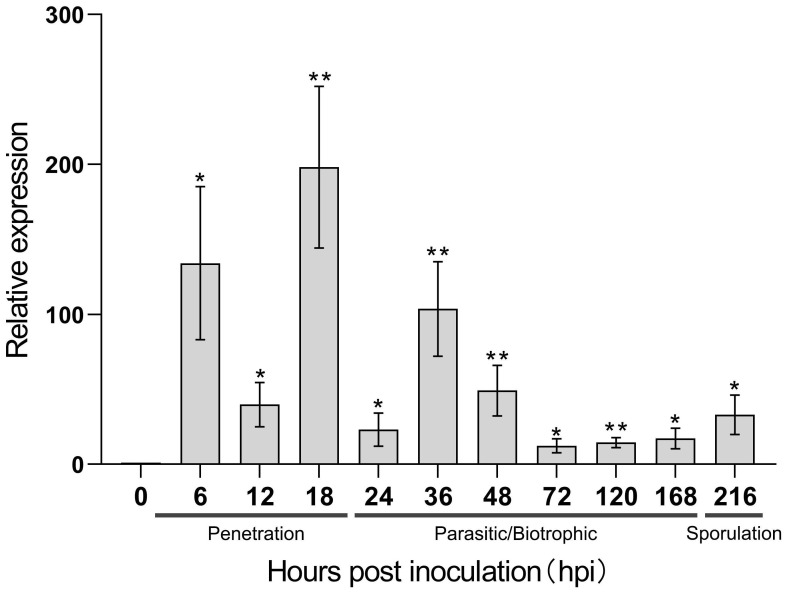
Transcriptional profiles of *PstChia1* during wheat-*Pst* interaction. After inoculation with CYR31, leaf samples were collected at 0, 6, 12, 18, 24, 36, 48, 72, 120, 168, and 216 hpi, respectively, and three biological replicates were calculated using the comparison threshold (2^−ΔΔCT^) method. By the *T*-test, an asterisk indicates a significant difference at the same time point (* *p* < 0.05, ** *p* < 0.01).

For further study, we selected *PstChia1*, the most highly expressed during wheat and *Pst* interaction, for qRT-PCR analysis (Figure 3). Our results showed that *PstChia1* was highly induced at two time points. The first was at the very early stage of spore germination and colonization, reaching a 134-fold increase at 6 hpi and then down-regulated to 39-fold at 12 hpi. The second peak value was in the early stage of haustorium formation, reaching the highest expression level of a 198-fold increase during the entire infection period from 18 hpi before the expression decreased (Figure 3). Those results suggested that *PstChia1* may play an important role in the early stages of infection.

### 2.3. *PstChia1* Has Chitinase Activity

**Figure 4:**
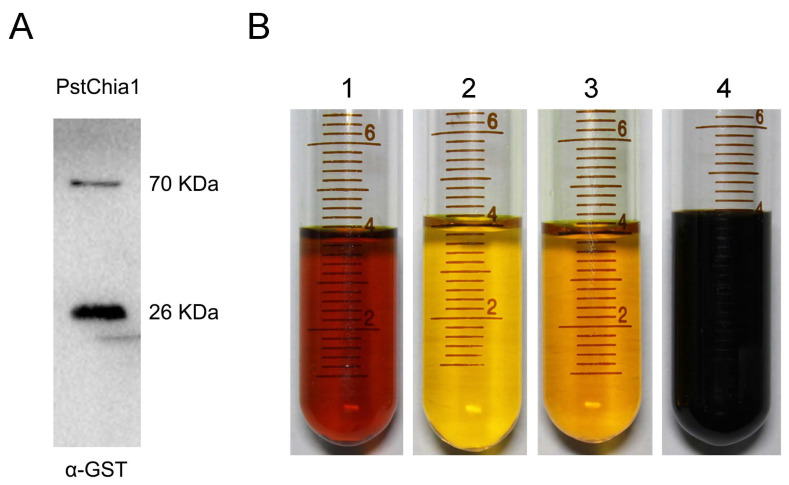
Enzyme activity analysis of *PstChia1*. (**A**) Western blot results of *PstChia1*. An antibody against GST was used. (**B**) The results of the DNS chromogenic reaction of *PstChia1*. 1. Group containing *PstChia1-GST* protein with 0.5% colloidal chitin; 2. Group containing the GST-tag protein with 0.5% colloidal chitin; 3. Group containing only 0.5% colloidal chitin; 4. Group containing 0.5% GlcNAc.

The protein in the supernatant solution was obtained by prokaryotic expression of chitinase gene *PstChia1-GST*, and the protein expression was verified by Western blot. According to the prediction of the amino acid sequence of the chitinase gene *PstChia1*, the relative analytical quantity of this protein was about 44 kDa. The GST-tag was about 26 kDa. The result confirmed that the protein in the supernatant is the target protein of the *PstChia1-GST*, being 70 kDa (Figure 4A). Chitin degradation was determined by the DNS method. Four different treatments were included in this experiment. Group 1 combined *PstChia1-GST* protein with 0.5% colloidal chitin. Group 2 combined the GST-tag protein with 0.5% colloidal chitin. Group 3 contained only 0.5% colloidal chitin. Group 4 contained only 0.5% GlcNAc. As shown in Figure 4B, the first and the fourth groups were becoming deep red, and the second and third groups did not change. This result indicated that *PstChia1-GST* degraded chitin and produced reducing sugar GlcNAc (Figure 4B).

### 2.4. Silencing of *PstChia1* Reduces the Pathogenicity of Pst

**Figure 5:**
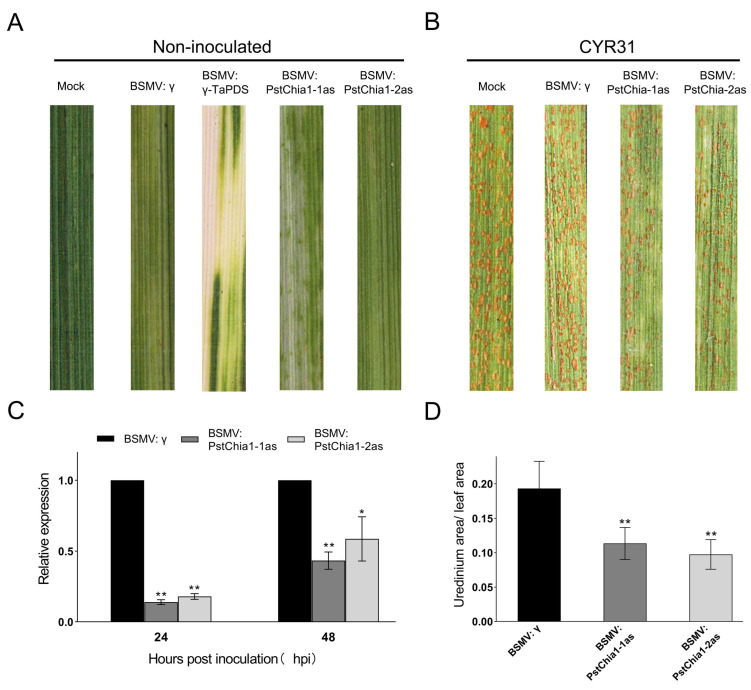
BSMV-HIGS functional characterization of *PstChia1*. (**A**) BSMV: γ-TaPDS showed photobleaching at 10 dpi; Mock: Wheat leaves treated with 1X fes buffer. (**B**) The fourth leaf of the *Pst* isolate CYR31. Leaves were photographed at 14 dpi. (**C**) Evaluation of the silencing effect of knockout in plants inoculated with the *Pst* isolate CYR31. (**D**) Relative uredinium area of *Pst* in wheat seedlings. Three biological replicates were calculated using the comparison threshold (2^−ΔΔCT^) method. Asterisks indicate a significant difference at the same point (* *p* < 0.05, ** *p* < 0.01).

To further characterize *PstChia1* in *Pst*, we knocked down the *PstChia1* using Barley Stripe Mosaic Virus (BSMV)-mediated host-induced gene silencing. Two silencing fragments of the *PstChia1* gene, designated *PstChia1-1as* and *PstChia1*-*2as*, were used to generate virus constructs, respectively (Primers are listed in Table S4). The BSMV: γ-TaPDS showed obvious photobleaching, and all of the wheat leaves infected with BSMV: γ, BSMV: *PstChia1-1/2as* expressed similar phenotypes of mild chlorotic mosaic symptoms at 10 d post-inoculation (Figure 5A). The fourth leaves of wheat plants, which were inoculated with BSMV, were then inoculated with the fresh urediniospores of virulent isolate CYR31. The number of rust pustules was reduced in the leaves inoculated with BSMV: *PstChia1-1/2as* compared with the leaves of BSMV: γ inoculated wheat (Figure 5B). The relative expression examined by qRT-PCR, the expression levels of *PstChia1-1as* and *PstChia1*-*2as* knockdown plants were significantly reduced compared with the control at 24 hpi and 48 hpi, indicating that *PstChia1* was successfully silenced (Figure 5C). Compared with the negative control BSMV: γ, the *Pst* infection area was significantly decreased in the leaves inoculated with BSMV: *PstChia1-1/2as* (Figure 5D). These results indicated that *PstChia1* contributed to the virulence of *Pst*.

### 2.5. Knockdown of *PstChia1* Inhibits the Development of Pst

**Figure 6:**
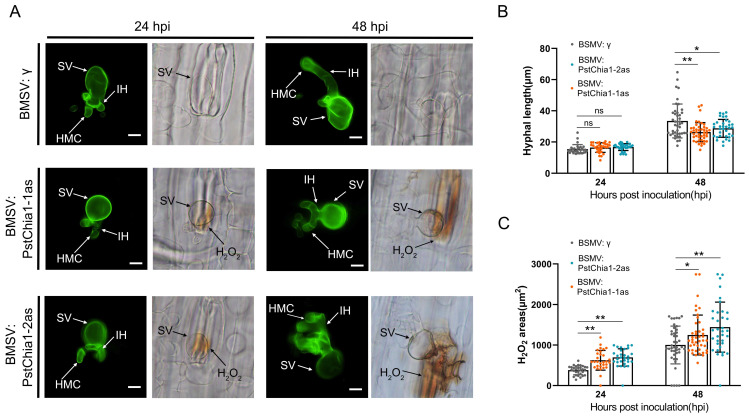
Histological observation of H_2_O_2_ and fungal development. (**A**) The silenced leaves were inoculated with virulent race CYR31. H_2_O_2_ around the infected area was observed by 3,3-diaminobenzidine (DAB) staining at 24 and 48 hpi, and the leaves were stained with wheat germ agglutinin (WGA) to reveal the pathogen at 24 and 48 hpi. (**B**) Hyphal length was measured at 24 and 48 hpi. (**C**) Area of H_2_O_2_ was measured at 24 and 48 hpi. Asterisks indicate a significant difference (* *p* < 0.05, ** *p* < 0.01). SV, substomatal vesicles; IH, infection hypha; HMC, haustorium mother cell. The data were collected from 30 sites of infection. Hpi, h after inoculation; Bar, 10 µm.

In order to analyze the effect of *PstChia1-1as* and *PstChia1-2as* knockdown wheat plants on the growth of *Pst*, samples were collected at 24 h and 48 h after inoculation with virulent CYR31. Histological observation showed that the hyphal length of the *PstChia1-1as* and *PstChia1-2as* knockdown plants was significantly reduced at only 48 hpi (Figure 6A,B). In addition, the accumulation of H_2_O_2_ around the infection sites was significantly induced at 24 and 48 hpi (Figure 6A,C). These results suggest that the knockdown of *PstChia1* influences the development of *Pst*.

### 2.6. Defense-Related Genes Were Influenced by Knockdown of PstChia1

**Figure 7:**
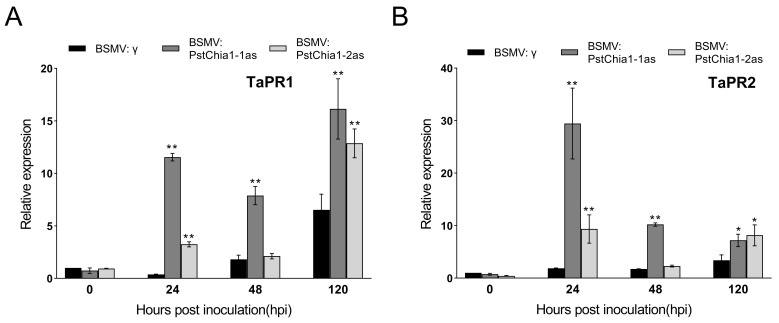
Transcript profiles of pathogenesis-related (PR) genes in *PstChia1* knockdown plants. (**A**) The expression level of TaPR1. (**B**) The expression level of TaPR2. Asterisks indicate a significant (* *p* < 0.05, ** *p* < 0.01).

To investigate disease resistance in *PstChia1* knockdown plants, the expression level of pathogenesis-related (PR) genes was examined by RT-qRCR. Our results showed that the relative expression of TaPR1 and TaPR2 were significantly upregulated in both *PstChia1* and *PstChia2* knockdown plants (Figure 7A,B).

## 3. Discussion

Chitin, one of the most abundant biopolymers, is the main structural component of fungal cell walls. Chitinases hydrolyze the beta-1,4-linkages of chitin [32,33]. Fungal chitinases play an important role during nutritional chitin acquisition, competition with other fungi or arthropods, and cell wall remodeling, including hyphal growth, branching, hyphal fusion, and autolysis [34,35]. Chitinases are grouped into glycosyl hydrolases 18 (GH18) and glycosyl hydrolases 19 (GH19) families [36]. The GH18 family is widely distributed in viruses, bacteria, plants, animals, and fungi, while the GH19 family is found mainly in plants, with some reports in viruses, bacteria, and nematodes [37,38,39]. To date, all the fungal chitinases belong to the GH18 family, with a wide variation in the number of genes. For example, only a single chitinase gene was found in *Schizosaccharomyces pombe*, while 36 chitinase genes were identified in *Trichoderma virens* [40]. In this study, we analyzed the chitinase gene family in 10 fungal genomes (five in Ascomycota and five in Basidiomycota). The number of chitinase genes ranges from two in *Saccharomyces cerevisiae* to 19 in *Verticillium dahlia* (Figure 1 and Table S1). In general, the number of chitinase genes in yeast is less than the number in filamentous fungi. By contrast, filamentous fungi expand the number of chitinase genes and indicate a functional redundancy, which is a challenge for investigating chitinase research involving multiple deletion mutants [35]. Notably, the plant pathogen *Ustilago maydis* has four chitinases (three were reported) and belongs to different clusters, which facilitates functional analysis (Figure 1) [41,42].

Chitinases must be secreted in order to act on the cell wall [43]. Most of the secreted proteins have the N-terminal signal peptide and are processed through the ER and Golgi apparatus with post-translational modifications for conventional secretion. However, some cell wall-associated proteins do not have the signal peptides, suggesting that they have an unconventional secretory pathway [44,45]. Similarly, we identified 131 chitinase genes in 10 fungal genomes, and only 65 of them contained signal peptides. Nearly half of the fungal chitinases contain the signal peptide, implying that those chitinases may have evolved from two types of ancient genes. The chitinases UmCts1 in *U. maydis*, ScCts2p in *S. cerevisiae*, and CaCht4p in *Candida albicans* have been reported as secreted proteins without a signal peptide [44,45,46,47]. Interestingly, the UmCts1 and UmCts2 (with a signal peptide) have redundant functions in cell separation [42]. Thus, it is likely that the chitinases were employed via different secretory pathways.

The regulation of fungal chitinases varies with their different functions. Housekeeping chitinases are constitutively expressed and thought to participate in continuous cell wall remodeling [35]. The gene *gh18-10* in *Neurospora crassa* is constitutively expressed during growth. Mutation of this housekeeping chitinase gene reduced fungal growth [48]. In *Pst*, *PstChia6*, *PstChia7*, and *PstChia8* are constitutively expressed during compatible and incompatible interactions (Figure 2A). The phylogenetic tree shows that *PstChia6* and *PstChia7* are members of Cluster III and have a close relationship with the chitinases of Ascomycota (Figure 1). However, *PstChia8* clusters in Cluster Ib with most Basidiomycota chitinases and is the only chitinase that has a PNGaseA domain in the C-terminal (Figure 1 and Figure 2B). This result implies that *PstChia6*, *PstChia7*, and *PstChia8* may be housekeeping chitinases, whereas *PstChia8* may have an additional function in cleaving glycopeptides.

Chitin is an important immunity activator during infection by filamentous fungi. Some chitinase genes are highly induced during infection and have been reported as the effector to suppress chitin-triggered immunity, such as the MpChi from *Moniliophthora pernicosa*, MoChia1 from *Magnaporthe oryzae*, or the EWCAs from *Podosphaera xanthii* [27,28,49]. Those studies demonstrated that fungi evolved new functions of chitinase. In our study, RNA-seq data indicated that the expression of *PstChia1* was the highest among the chitinases in both compatible and incompatible interactions of wheat and *Pst*. Furthermore, the relative expression showed that *PstChia1* was the most highly induced at 18 hpi in the penetration stage of *Pst* infection (Figure 3). Analysis of the enzyme activity showed that *PstChia1* is enzymatically active (Figure 4). Those results suggested that *PstChia1* may play an important role during the wheat-*Pst* interaction.

BMSV-mediated HIGS is an efficient tool for studying the gene function of obligate pathogens. For example, silencing of *Pst18363* and CYP51 was shown to attenuate the pathogenicity of *Pst* [50]. In this study, silencing of *PstChia1* silenced by HIGS resulted in a decreased number of uredinia. In the *PstChia1*-knockdown plant, the length of infection hyphae was also significantly reduced. Furthermore, the expression of two defense marker genes, *TaPR1* and *TaPR2*, was induced in the *PstChia1* knockdown plants. Those results suggest that *PstChia1* contributes to pathogenicity by interfering with plant immunity and regulating the morphology and growth of *Pst*. Recently, many studies have demonstrated that effectors can target and influence the host genes to regulate the host immune responses [51]. Thus, we hypothesize that *PstChia1* may also target wheat genes to decrease the plant immunity response. Further, the stable transgenic wheat lines of the *PstChias* need to be produced, and the molecular mechanisms by which *PstChia1* suppresses the wheat defense response and the genes that interact with it will be a major focus of our further investigations.

Overall, we identified 16 chitinase genes in *Pst* and analyzed their expression patterns during the *Pst* infection stages. We first demonstrated that *PstChia1*, one of the chitinase proteins in *Pst*, contributed to pathogenicity by disturbing plant immunity. This is new evidence to promote a better understanding of chitinase proteins in biotrophic fungi, their role in pathogenesis, and the molecular basis of defense suppression that may reveal a candidate gene for wheat resistance breeding by HIGS.

## 4. Materials and Methods

### 4.1. Identification and Phylogenetic Analysis of PstChia Genes

The conserved domain GH18 (PF00704) of the chitinase gene was used as a seed to compare 33 fungal genome sequences to obtain candidate chitinase genes in HMMER 3.0. Criteria (E < 10^−5^) were used to ensure the reliability of the protein sequences. These candidate genes were subsequently submitted to the Pfam31.0 (http://pfam.xfam.org/, accessed on 20 December 2022) database to further confirm that all sequences contained the GH18 conserved domain. The phylogenetic relationship was inferred with the Maximum Likelihood (ML) method based on the LG model [52] in MEGA6.0 [53]. The midpoint rooted base tree was drawn using Interactive Tree of Life (IToL) Version 6.7.1. Scale bars correspond to 0.5 amino acid substitutions.

### 4.2. Analysis of Gene Expression

The transcript levels of all *PstChia* genes were determined by time series dual RNA-Seq data in our lab. We sequenced two groups of wheat-*Pst* interaction combinations, named NIL_R vs. CYR32 and NIL_S vs. CYR32, and selected the time points at 0, 18, 24, 48, 96, and 168 hpi. The wheat cultivar NIL_R (Yr26) and the susceptible line NIL_S (yr26) were generated by 92R137 (Yr26 gene donor) backcross with recurrent parent Yangmai 158 six times and self-cross four times (BC6F4) [54]. A single spore isolate of CYR32 was reproduced on seedlings of wheat cultivar Mingxian169. The fresh urediniospores were collected and used for inoculating. NIL_S vs. 32R was a compatible group (wheat is susceptible to rust), while NIL_R vs. CYR32 was an incompatible group. However, the compatible group and the incompatible group were simply named 32S and 32R, respectively. Each sample was sequenced 10 Gb on HiSeq2500 (PE125) and mapped to Chinese spring (TGACv1) [55] and CYR32 [56] reference (Accession number CNSA: CNP0001524, https://db.cngb.org/cnsa/, accessed on 20 December 2022). The transcriptome data were used to analyze the expression of all *PstChia* genes, and TBtools was used for the heatmap [57].

### 4.3. Analysis of Gene Transcriptional Levels by Quantitative Real-Time PCR

To evaluate the transcript levels of *PstChia1* in response to CYR31 infection, wheat leaves were sampled at 0, 6, 12, 18, 24, 36, 48, 72, 120, 168, and 216 hpi based on previous microscopic observations of the wheat–*Pst* interaction [58]. The Quick RNA Isolation Kit (Huayueyang Biotechnology, China, Beijing) was used to extract RNA from all samples. The RNA was reversed transcribed to cDNA by the RevertAid First Strand cDNA Synthesis Kit (Fermentas, Waltham, MA, USA). Quantification of gene transcriptional levels was performed with the CFX Connect Real-Time PCR System (Bio-Rad, Hercules, CA, USA). The *Pst* translation elongation factor 1 (PsEF1) gene was used as the internal reference for normalization [59]. The transcript levels of the *PstChia1* gene in this study were assayed by the comparative 2^−ΔΔCT^ method [60].

### 4.4. Enzyme Activity Assay of Chitinase PstChia1

The prokaryotic expression vector *PstChia1-pGEX4T-1* (containing a GST tag for protein expression) was constructed and transformed into *E. coli* strain BL21. The cells were harvested by centrifugation, resuspended in ice-cold phosphate-buffered saline (PBS), and lysed by ultrasonic fragmentation in accordance with the supplier’s instructions of the ultrasonic instrument (SCIENTZ, Ningbo, China; Amplitude 30%, Pulse on 4 s, Pulse off 6 s, total time 15 min). The cell lysate was centrifuged at 8000 rpm for 20 min at 4 degrees. The supernatant was removed, and SDS sample loading buffer was added into the supernatant containing the *Gst-PstChia1* fusion proteins and heated to 100 degrees for 5 min for Western blot analysis. We used Mini-PROTEAN Tetra Cell Casting Module (Bio-Rad, Hercules, CA, USA) to hand cast PAGE polyacrylamide gels according to the manufacturer’s protocol of the one-step PAGE gel fast preparation kit (Epizyme, Shanghai, China). The proteins were separated by PAGE using running buffer (200-mM Glycine, 25-mM Tris, 0.1% SDS [*w*/*v*]) in a mini-protean tetra electrophoresis cell (Bio-Rad, Hercules, CA, USA) and the gel was blotted onto a PVDF membrane (Merck Millipore, Burlington, MA, USA) using transfer buffer (200-mM Glycine,25-mM Tris) in a mini-protean tetra trans-blot cell (Bio-Rad, Hercules, CA, USA) at 65 V. The membrane was blocked for 1 h at room temperature using blocking solution (TBST containing 5% skimmed milk powder [*w*/*v*]). The anti-GST antibody (1:1000; #66001-2-Ig, Proteintech, Sankt Leon-Rot, Germany) was added, and the membranes were incubated at 4 degrees overnight, followed by three washes using TBST (0.02% KCl [*w*/*v*], 0.3% Tris [*w*/*v*], 0.8% NaCl [*w*/*v*]). The membrane was then incubated with goat anti-mouse antibody (1:1000; #A0192, Beyotime) at room temperature for 1 h. After washing three times, the membrane was incubated with Monpro ECL ultrasensitive substrate pro (1:1, #PW30701S, monad) for 5 min and visualized by excitation at 780 or 800 nm.

Due to *PstChia1* containing the conserved domain GH18 of chitinase, it is speculated that *PstChia1* had the function of hydrolyzing chitin into reducing sugar GlcNAc. Previous studies have shown that a discoloration reaction occurs when a mixture of 3,5-dinitrosalicylic acid (DNS) is heated in a boiling water bath for 5 min with reducing sugars under alkaline conditions [61]. Based on this principle, the purified target protein and GST protein could be mixed with colloidal chitin, respectively, and the control was used with GlcNAc and colloidal chitin without proteins. The four treatments were treated at 4 °C for 24 h, and the changes of color were observed by adding DNS solution and heating to 100 degrees for 5 min.

### 4.5. BSMV Mediate Host-Induced Gene Silencing

Two specific-sequence fragments were selected for *PstChia1* and *PstChia1-1/2as*, and primers were designed using Prime 5 and evaluated by BLASTN searches in the NCBI database. Analysis showed that these two fragments were specific for silencing these genes. Two fragments of these genes were cloned and transferred into barley stripe mosaic virus (BSMV) to produce BSMV: *PstChia1-1/2as*. The plasmids, which had been fully linearized in the previous step, were transcribed in vitro according to the instructions for the RiboMAXTM Large Scale RNA Production Systems-T7 kit. Fes buffer and BSMV: γ were inoculated as a negative control and BSMV: TaPDS as a positive control. Plant cultivation and virus inoculation were carried out as before [62]. Inoculation with the virulent race, CYR31, was performed 10 days after virus inoculation; the fourth leaf was sampled at 24 and 48 hpi to measure silencing efficiency and for histological observations. Fourteen days after inoculation with *Pst*, the onset of symptoms was observed and recorded, and the phenotype was photographed. These experiments were repeated three times.

### 4.6. Histological Observations of Fungal Growth and H_2_O_2_ Accumulation

Wheat leaves inoculated with *Pst* were sampled and stained at 24 h and 48 h to observe H_2_O_2_ accumulation and hyphal growth. H_2_O_2_ accumulation was detected by staining with the 3,3-diaminobenzidine (DAB) and observed under white light [63]. *Pst* infection structures were stained with wheat germ agglutinin (WGA), and the stained tissue was observed under blue light excitation (excitation wavelength 450~480 nm, emission wavelength 515 nm) [64]. The stained tissue was decolorized in a solution of anhydrous ethanol and glacial acetic acid (1:1). H_2_O_2_ accumulation and fungal structures such as hyphae, substomatal vesicles, and haustoria around the infection sites were observed with a BX-51 microscope (Olympus, Tokyo, Japan), and their corresponding lengths and areas were estimated using DP-BSW software. More than five leaves were randomly selected for observation of each treatment group, and 50 relevant areas were calculated.

### 4.7. Analysis of RT-PCR Results for Pathogenesis-Related Protein Genes

To determine whether silencing *PstChia1* affects the expression of PR genes in wheat, *PstChia1*-*1as* and *PstChia1*-*2as* were constructed into viral vector γ, respectively. The constructed vectors were inoculated with the virus on wheat after linearization and in vitro transcription to RNA. The vector and the negative and positive controls were identical to those of the previous VIGS, and more detailed methods for gene silencing, vector construction, and virus inoculation were followed from previous studies [65]. Sampling was carried out at 24, 48, and 120 h after inoculation with *Pst*, and Real-time PCR was used to measure the expression of the disease process-related protein genes TaPR1 and TaPR2 after inoculation of plants with *PstChia1*.

## Data Availability

The data presented in this study are available on request from the corresponding author.

## Supplementary Materials

The supporting information can be downloaded at: https://www.mdpi.com/article/10.3390/ijms24098215/s1.

## References

[1] Jones J.D., Dangl J.L. (2006). The plant immune system. Nature.

[2] Chen H., Lin Y., Tu S., Wang L., Chen Z., Zeng M., Xiao J., Yuan P., Qiu M., Wang Y. (2023). The *Phytophthora sojae* nuclear effector PsAvh110 targets a host transcriptional complex to modulate plant immunity. Plant Cell.

[3] Yu X., Feng B., He P., Shan L. (2017). From chaos to harmony: Responses and signaling upon microbial pattern recognition. Annu. Rev. Phytopathol..

[4] Albert I., Hua C., Nürnberger T., Pruitt R.N., Zhang L. (2020). Surface sensor systems in plant immunity. Plant Physiol..

[5] Zhou J.-M., Zhang Y. (2020). Plant Immunity: Danger perception and signaling. Cell.

[6] Cui H., Tsuda K., Parker J.E. (2015). Effector-triggered immunity: From pathogen perception to robust defense. Annu. Rev. Plant Biol..

[7] Thomma B.P., Nürnberger T., Joosten M.H. (2011). Of PAMPs and effectors: The blurred PTI-ETI dichotomy. Plant Cell.

[8] Boller T., Felix G. (2009). A renaissance of elicitors: Perception of microbe-associated molecular patterns and danger signals by pattern-recognition receptors. Annu. Rev. Plant Biol..

[9] Zipfel C. (2014). Plant pattern-recognition receptors. Trends Immunol..

[10] Zipfel C., Kunze G., Chinchilla D., Caniard A., Jones J.D., Boller T., Felix G. (2006). Perception of the bacterial PAMP EF-Tu by the receptor EFR restricts agrobacterium-mediated transformation. Cell.

[11] Gaulin E., Drame N., Lafitte C., Torto-Alalibo T., Martinez Y., Ameline-Torregrosa C., Khatib M., Mazarguil H., Villalba-Mateos F., Kamoun S. (2006). Cellulose binding domains of a *Phytophthora* cell wall protein are novel pathogen-associated molecular patterns. Plant Cell.

[12] Rotblat B., Enshell-Seijffers D., Gershoni J.M., Schuster S., Avni A. (2002). Identification of an essential component of the elicitation active site of the EIX protein elicitor. Plant J..

[13] Song T., Zhang Y., Zhang Q., Zhang X., Shen D., Yu J., Yu M., Pan X., Cao H., Yong M. (2021). The N-terminus of an *Ustilaginoidea virens* Ser-Thr-rich glycosylphosphatidylinositol-anchored protein elicits plant immunity as a MAMP. Nat. Commun..

[14] Ngou B.P.M., Ahn H.-K., Ding P., Jones J.D. (2021). Mutual potentiation of plant immunity by cell-surface and intracellular receptors. Nature.

[15] Yuan M., Jiang Z., Bi G., Nomura K., Liu M., Wang Y., Cai B., Zhou J.-M., He S.Y., Xin X.-F. (2021). Pattern-recognition receptors are required for NLR-mediated plant immunity. Nature.

[16] Ngou B.P.M., Ding P., Jones J.D. (2022). Thirty years of resistance: Zig-Zag through the plant immune system. Plant Cell.

[17] Debono M., Gordee R.S. (1994). Antibiotics that inhibit fungal cell wall development. Annu. Rev. Microbiol..

[18] Shibuya N., Minami E. (2001). Oligosaccharide signalling for defence responses in plant. Physiol. Mol. Plant Pathol..

[19] Kaku H., Nishizawa Y., Ishii-Minami N., Akimoto-Tomiyama C., Dohmae N., Takio K., Minami E., Shibuya N. (2006). Plant cells recognize chitin fragments for defense signaling through a plasma membrane receptor. Proc. Natl. Acad. Sci. USA.

[20] Miya A., Albert P., Shinya T., Desaki Y., Ichimura K., Shirasu K., Narusaka Y., Kawakami N., Kaku H., Shibuya N. (2007). CERK1, a LysM receptor kinase, is essential for chitin elicitor signaling in *Arabidopsis*. Proc. Natl. Acad. Sci. USA.

[21] Cohen-Kupiec R., Chet I. (1998). The molecular biology of chitin digestion. Curr. Opin. Biotechnol..

[22] Hadwiger L.A., Beckman J.M. (1980). Chitosan as a component of Pea-*Fusarium Solani* interactions. Plant Physiol..

[23] Nasser W., de Tapia M., Kauffmann S., Montasser-Kouhsari S., Burkard G. (1988). Identification and characterization of maize pathogenesis-related proteins. Four maize PR proteins are chitinases. Plant Mol. Biol..

[24] Tyler L., Bragg J.N., Wu J., Yang X., Tuskan G.A., Vogel J.P. (2010). Annotation and comparative analysis of the glycoside hydrolase genes in *Brachypodium Distachyon*. BMC Genom..

[25] Zhang Y.-J., Ren L.-L., Lin X.-Y., Han X.-M., Wang W., Yang Z.-L. (2022). Molecular evolution and functional characterization of chitinase gene family in Populus Trichocarpa. Gene.

[26] Sánchez-Vallet A., Saleem-Batcha R., Kombrink A., Hansen G., Valkenburg D.-J., Thomma B.P., Mesters J.R. (2013). Fungal effector ecp6 outcompetes host immune receptor for chitin binding through intrachain LysM dimerization. eLife.

[27] Fiorin G.L., Sanchéz-Vallet A., de Toledo Thomazella D.P., do Prado P.F.V., do Nascimento L.C., de Oliveira Figueira A.V., Thomma B.P., Pereira G.A.G., Teixeira P.J.P.L. (2018). Suppression of plant immunity by fungal chitinase-like effectors. Curr. Biol..

[28] Martínez-Cruz J., Romero D., Hierrezuelo J., Thon M., de Vicente A., Pérez-García A. (2021). Effectors with chitinase activity (EWCAs), a family of conserved, secreted fungal chitinases that suppress chitin-triggered immunity. Plant Cell.

[29] Boller T., He S.Y. (2009). Innate immunity in plants: An arms race between pattern recognition receptors in plants and effectors in microbial pathogens. Science.

[30] Zipfel C. (2008). Pattern-recognition receptors in plant innate immunity. Curr. Opin. Immunol..

[31] Garnica D.P., Nemri A., Upadhyaya N.M., Rathjen J.P., Dodds P.N. (2014). The ins and outs of Rust Haustoria. PLoS Pathog..

[32] Gow N.R., Latge J.-P., Munro C.A., Heitman J., Howlett B.J., Crous P.W., Stukenbrock E.H., James T.Y., Gow N.A.R. (2017). The fungal cell wall: Structure, biosynthesis, and function. The Fungal Kingdom.

[33] Seidl V. (2008). Chitinases of filamentous fungi: A large group of diverse proteins with multiple physiological functions. Fungal Biol. Rev..

[34] Yang J., Zhang K.-Q. (2019). Chitin synthesis and degradation in fungi: Biology and enzymes. Targeting Chitin-Containing Organisms.

[35] Langner T., Göhre V. (2016). Fungal chitinases: Function, regulation, and potential roles in plant/pathogen interactions. Curr. Genet..

[36] Henrissat B. (1991). A classification of glycosyl hydrolases based on amino acid sequence similarities. Biochem. J..

[37] Duo-Chuan L. (2006). Review of fungal chitinases. Mycopathologia.

[38] Honda Y., Taniguchi H., Kitaoka M. (2008). A reducing-end-acting chitinase from vibrio *Proteolyticus* belonging to glycoside hydrolase family 19. Appl. Microbiol. Biotechnol..

[39] Geng J., Plenefisch J., Komuniecki P.R., Komuniecki R. (2002). Secretion of a novel developmentally regulated chitinase (family 19 glycosyl hydrolase) into the perivitelline fluid of the parasitic nematode, *Ascaris suum*. Mol. Biochem. Parasitol..

[40] Gruber S., Seidl-Seiboth V. (2012). Self versus non-self: Fungal cell wall degradation in *Trichoderma*. Microbiology.

[41] Koepke J., Kaffarnik F., Haag C., Zarnack K., Luscombe N.M., König J., Ule J., Kellner R., Begerow D., Feldbrügge M. (2011). The RNA-binding protein Rrm4 is essential for efficient secretion of endochitinase Cts1. Mol. Cell. Proteom..

[42] Langner T., Öztürk M., Hartmann S., Cord-Landwehr S., Moerschbacher B., Walton J.D., Göhre V. (2015). Chitinases are essential for cell separation in *Ustilago maydis*. Eukaryot. Cell.

[43] Heijne G.V. (1990). The signal peptide. J. Membr. Biol..

[44] Stock J., Sarkari P., Kreibich S., Brefort T., Feldbrügge M., Schipper K. (2012). Applying unconventional secretion of the endochitinase Cts1 to export heterologous proteins in *Ustilago maydis*. J. Biotechnol..

[45] Steringer J.P., Lange S., Čujová S., Šachl R., Poojari C., Lolicato F., Beutel O., Müller H.-M., Unger S., Coskun Ü. (2017). Key steps in unconventional secretion of fibroblast growth factor 2 reconstituted with purified components. eLife.

[46] Teparić R., Stuparević I., Mrša V. (2007). Binding assay for incorporation of alkali-extractable proteins in the *Saccharomyces cerevisiae* cell wall. Yeast.

[47] Chaffin W.L. (2008). Candida albicans cell wall proteins. Microbiol. Mol. Biol. Rev..

[48] Tzelepis G.D., Melin P., Jensen D.F., Stenlid J., Karlsson M. (2012). Functional analysis of glycoside hydrolase family 18 and 20 genes in *Neurospora crassa*. Fungal Genet. Biol..

[49] Yang C., Yu Y., Huang J., Meng F., Pang J., Zhao Q., Islam M.A., Xu N., Tian Y., Liu J. (2019). Binding of the *Magnaporthe oryzae* chitinase MoChia1 by a rice tetratricopeptide repeat protein allows free chitin to trigger immune responses. Plant Cell.

[50] Yang Q., Huai B., Lu Y., Cai K., Guo J., Zhu X., Kang Z., Guo J. (2020). A stripe rust effector Pst18363 targets and stabilises TaNUDX23 that promotes stripe rust disease. New Phytol..

[51] Lorrain C., Petre B., Duplessis S. (2018). Show Me the Way: Rust effector targets in heterologous plant systems. Curr. Opin. Microbiol..

[52] Le S.Q., Gascuel O. (2008). An improved general amino acid replacement matrix. Mol. Biol. Evol..

[53] Kumar S., Stecher G., Tamura K. (2016). MEGA7: Molecular evolutionary genetics analysis version 7.0 for bigger datasets. Mol. Biol. Evol..

[54] Wang C., Zhang Y., Han D., Kang Z., Li G., Cao A., Chen P. (2008). SSR and STS markers for wheat stripe rust resistance gene *Yr26*. Euphytica.

[55] Clavijo B.J., Venturini L., Schudoma C., Accinelli G.G., Kaithakottil G., Wright J., Borrill P., Kettleborough G., Heavens D., Chapman H. (2017). An improved assembly and annotation of the allohexaploid wheat genome identifies complete families of agronomic genes and provides genomic evidence for chromosomal translocations. Genome Res..

[56] Zheng W., Huang L., Huang J., Wang X., Chen X., Zhao J., Guo J., Zhuang H., Qiu C., Liu J. (2013). High genome heterozygosity and endemic genetic recombination in the wheat stripe rust fungus. Nat. Commun..

[57] Chen C., Chen H., Zhang Y., Tomas H.R., Frank M.H., He Y., Xia R. (2020). TBtools: An integrative toolkit developed for interactive analyses of Big Biological Data. Mol. Plant.

[58] Wang C.-F., Huang L.-L., Buchenauer H., Han Q.-M., Zhang H.-C., Kang Z.-S. (2007). Histochemical studies on the accumulation of reactive oxygen species (O^2−^ and H_2_O_2_) in the incompatible and compatible interaction of wheat—*Puccinia striiformis* f. sp. *tritici*. Physiol. Mol. Plant Pathol..

[59] Guo J., Dai X., Xu J.-R., Wang Y., Bai P., Liu F., Duan Y., Zhang H., Huang L., Kang Z. (2011). Molecular characterization of a Fus3/Kss1 type MAPK from *Puccinia striiformis* f. sp. *tritici*, PsMAPK1. PLoS ONE.

[60] Livak K.J., Schmittgen T.D. (2001). Analysis of relative gene expression data using real-time quantitative PCR and the 2^− ΔΔCT^ method. Methods.

[61] Miller G.L. (1959). Use of dinitrosalicylic acid reagent for determination of reducing sugar. Anal. Chem..

[62] Liu P., Duan Y., Liu C., Xue Q., Guo J., Qi T., Kang Z., Guo J. (2018). The calcium sensor TaCBL4 and its interacting protein TaCIPK5 are required for wheat resistance to stripe rust fungus. J. Exp. Bot..

[63] Thordal-Christensen H., Zhang Z., Wei Y., Collinge D.B. (1997). Subcellular localization of H_2_O_2_ in Plants. H_2_O_2_ accumulation in papillae and hypersensitive response during the barley—Powdery mildew interaction. Plant J..

[64] Bai X., Zhan G., Tian S., Peng H., Cui X., Islam M.A., Goher F., Ma Y., Kang Z., Xu Z.-S. (2021). Transcription factor BZR2 activates chitinase Cht20.2 transcription to confer resistance to wheat stripe rust. Plant Physiol..

[65] Liu P., Guo J., Zhang R., Zhao J., Liu C., Qi T., Duan Y., Kang Z., Guo J. (2019). TaCIPK10 interacts with and phosphorylates TaNH2 to activate wheat defense responses to stripe rust. Plant Biotechnol. J..

